# The Effects of Handling and Anesthetic Agents on the Stress Response and Carbohydrate Metabolism in Northern Elephant Seals

**DOI:** 10.1371/journal.pone.0038442

**Published:** 2012-05-31

**Authors:** Cory D. Champagne, Dorian S. Houser, Daniel P. Costa, Daniel E. Crocker

**Affiliations:** 1 Department of Ecology and Evolutionary Biology, University of California, Santa Cruz, California, United States of America; 2 Department of Biology, Sonoma State University, Rohnert Park, California, United States of America; Centre of Marine Sciences & University of Algarve, Portugal

## Abstract

Free-ranging animals often cope with fluctuating environmental conditions such as weather, food availability, predation risk, the requirements of breeding, and the influence of anthropogenic factors. Consequently, researchers are increasingly measuring stress markers, especially glucocorticoids, to understand stress, disturbance, and population health. Studying free-ranging animals, however, comes with numerous difficulties posed by environmental conditions and the particular characteristics of study species. Performing measurements under either physical restraint or chemical sedation may affect the physiological variable under investigation and lead to values that may not reflect the standard functional state of the animal. This study measured the stress response resulting from different handling conditions in northern elephant seals and any ensuing influences on carbohydrate metabolism. Endogenous glucose production (EGP) was measured using [6-^3^H]glucose and plasma cortisol concentration was measured from blood samples drawn during three-hour measurement intervals. These measurements were conducted in weanlings and yearlings with and without the use of chemical sedatives—under chemical sedation, physical restraint, or unrestrained. We compared these findings with measurements in adult seals sedated in the field. The method of handling had a significant influence on the stress response and carbohydrate metabolism. Physically restrained weanlings and yearlings transported to the lab had increased concentrations of circulating cortisol (F_11, 46_ = 25.2, p<0.01) and epinephrine (F_3, 12_ = 5.8, p = 0.01). Physical restraint led to increased EGP (t = 3.1, p = 0.04) and elevated plasma glucose levels (t = 8.2, p<0.01). Animals chemically sedated in the field typically did not exhibit a cortisol stress response. The combination of anesthetic agents (Telazol, ketamine, and diazepam) used in this study appeared to alleviate a cortisol stress response due to handling in the field without altering carbohydrate metabolism. Measures of hormone concentrations and metabolism made under these conditions are more likely to reflect basal values.

## Introduction

Free-ranging animals often cope with fluctuating environmental conditions such as weather, food availability, predation risk, the requirements of breeding, and the influence of anthropogenic factors. An animal's response to perturbation is, in large part, mediated by stress hormones (e.g. cortisol & epinephrine) [1]. These hormones have strong impacts on energy balance and metabolism, especially the maintenance of blood glucose levels [2], [3]. Thus, glucocorticoid concentrations have been correlated with food availability [4], increased feeding behavior [5], human disturbance [6]–[8], and survival [9]–[11]. Consequently, researchers are increasingly attempting to measure stress markers, especially glucocorticoids (e.g. cortisol), to understand stress, disturbance, and health in free-ranging populations [6], [12]–[14].

Studying free-ranging animals comes with numerous difficulties posed by environmental conditions and the particular characteristics of study species. Some tissues may be collected without animal handling (e.g. hair and feces) for glucocorticoid measurement [15], [16]. Ideally, however, researchers gather information not only on the indicators of stress but also a measure of physiological state (e.g. energy expenditure or metabolism) across multiple life-history stages [17]. These measures of metabolism in free-ranging animals, however, can be challenging. The doubly-labeled water method revolutionized the measurement of metabolic rate in free-ranging animals [18], [19] while advances in instrument technology now allow for the remote measurement of foraging behaviors [20]–[22] as well as estimates of metabolic rate by heart rate [23], [24] or accelerometry data [25]. For some studies, physiological measurements can be made in free-ranging animals by temporarily implanting probes and attaching recording devices [26]. In each of these cases, some degree of animal handling is required to investigate vital function in free-ranging animals. Usually, samples can only be collected after either physically restraining the animal or using chemical sedatives—both have potential confounding effects on the measured parameters. To counteract these stress artifacts researchers typically attempt to minimize any stress response due to capture by re-assessing and adjusting handling protocols [27]–[29]. For example, corticosterone measurements from blood samples collected within 2–3 minutes of capture probably reflect basal conditions in many bird species [30]. Conversely, studying an animal's response to a capture-stress protocol can provide insight into the individual's ability to cope with stressors [31].

For some study objectives and select physiological variables, transient stress responses to handling may not be important sources of measurement artifact. Since the hormones released during a stress response impact metabolic pathways [32], [33] investigations of whole-animal metabolism may be particularly sensitive to artifacts of stress responses. For example, acute stress responses result in increased levels of glucocorticoids and catecholamines, which affect the release of glucose into plasma [34]. Thus, studies of fuel metabolism are potentially influenced by stress artifacts from handling. These stress responses may be most quickly observed in carbohydrate metabolism, which is normally tightly regulated [35]. A variety of studies in free-ranging animals, including investigations of carbohydrate metabolism, e.g. [36], [37]–[42], and static measures of metabolites and hormones, e.g. [43], [44]–[47], are potentially impacted by responses to handling and sampling. Few studies, however, have quantitatively examined the impact of handling, chemical immobilization, or stress on glucose metabolism in wildlife.

The aim of this study was to measure the stress response from handling and sedation and determine its influence on physiological parameters (e.g. plasma glucose concentration and the rate of glucose production and use). We compared the metabolic responses to handling and restraint using standard metabolic tracer techniques to measure endogenous glucose production (EGP) and radioimmuno assay (RIA) to measure hormone levels in a well-studied species, the northern elephant seal (*Mirounga angustirostris*). We investigated the variability in metabolic and endocrine responses to capture and handling among four age classes: weaned pups, yearlings, adult females, and adult males. In one year, measurements were conducted under experimentally manipulated handling conditions—1) chemically sedated, 2) physically restrained, and 3) unrestrained seals. The response to handling in these controlled conditions was then compared with measurements conducted in the field under chemical sedation.

## Methods

### Study Design & Experimental Groups

Animals were studied during natural fasts while hauled-out at Año Nuevo state park (San Mateo county, CA) and included four age classes—weaned pups, yearlings, adult females, and adult males; these broad age classes are easily identified by size and pelage coloration. The study design and measurement conditions for each group are summarized in Table 1. There were two separate study groups: the *field sedated* and *handling manipulated* groups. Field sedated animals were only investigated while under chemical sedation at the field site and included weaned pups, adult females, and adult males. In the handling manipulated group, measurements were conducted under three experimental conditions—1) chemically sedated, 2) physically restrained, and 3) unrestrained. The handling manipulated group was composed of weanlings and yearlings. Measurements were made in weanlings in the field under chemical sedation and while physically restrained. Yearlings were studied while chemically sedated and while unrestrained but confined within a transport cage (see below for details). Under each experimental condition, EGP was measured over a 150–180 minute sampling period. Blood samples were drawn periodically for subsequent analysis of cortisol concentration in all study animals and plasma glucose and epinephrine in a subset of study animals.

**Table 1:** Summary of the experimental design and treatment groups used in this study.

Age Class	Study Year	Animal State	Restraint Type	n
**Handling Manipulated Group**				
Weanling	2008	mid postweaning fast	chemical sedation	5
			physical restraint	5
Yearling	2008	late molting	chemical sedation	7
			unrestrained	6
**Field Sedated Group**				
Weaned Pup	2003	early postweaning fast	chemical sedation	5
		late postweaning fast	chemical sedation	5
Adult Female	2003	early lactation	chemical sedation	5
		late lactation	chemical sedation	7
		late molting	chemical sedation	6
Adult Male	2007	early breeding	chemical sedation	5
		late breeding	chemical sedation	5
		late molting	chemical sedation	5
			**total:**	**66**

Measurements were made in four age classes at various times during natural fasts. This study used data from 46 elephant seals and reports cortisol responses for 66 procedures. Samples were collected in three separate years: 2003, 2007, and 2008. The handling manipulation measurements were made in 2008 on weanlings and fully molted yearlings both fasting for approximately 3–4 weeks. Using these handling manipulated animals, we tested the effects of restraint in a paired sample design. To make measurements in an unrestrained condition, yearlings were transported to the animal holding facility at Sonoma State University for both chemical sedation and unrestrained measurements. All other procedures were conducted in the field. Field sedated study groups consisted of weaned pups, measured early and late in their post-weaning fast (less than 2 weeks and over 6 weeks after weaning); adult females were measured early (5 days post-partum) and late in lactation (23 days post-partum). Late molting measurements, of both adult males and females, were made in fully molted animals with estimated fasting durations of 3–4 weeks. Breeding season measurements were made in adult males early (fasting less than 3 weeks) and late (fasting over 2 months) in the season.

### Treatment Procedures

**Figure 1:**
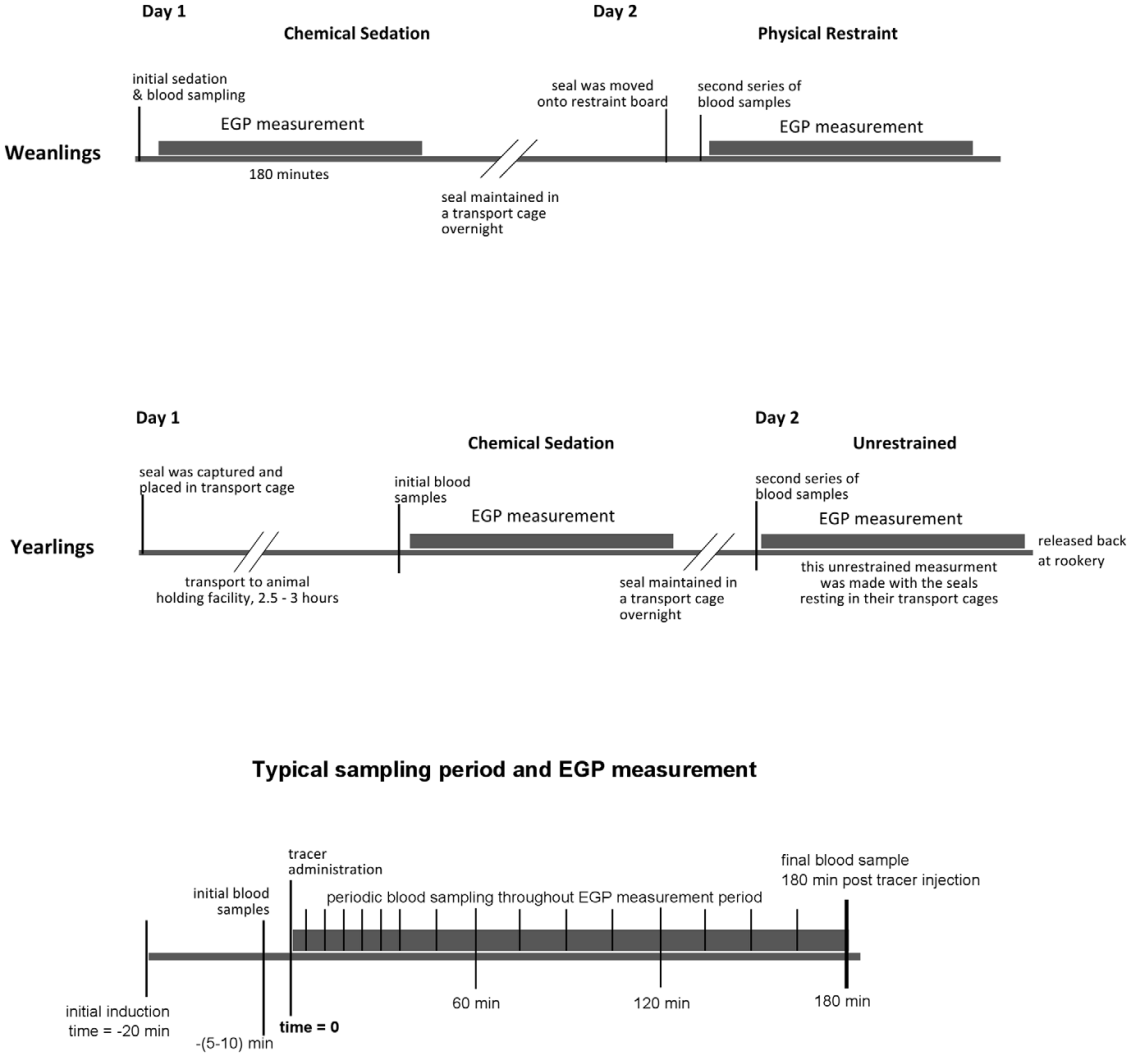
Summary of the treatment procedures used in this study. The measurement of endogenous glucose production (EGP) was performed by administering [6-^3^H]glucose as a tracer and periodically drawing blood samples over 180 minutes. Animals in the “field sedated” study group were all measured at the rookery. Weanlings and yearlings in the “handling manipulation” group were each measured twice over two consecutive days. Weanlings were studied at the rookery while yearlings were transported to an animal holding facility for study.

A summary and timeline of treatment procedures is shown in Figure 1. All procedures were approved by the Institutional Animal Care and Use Committee of Sonoma State University and conducted in accordance with the *Guide for the Care and Use of Laboratory Animals* published by the National Research Council (www.nap.edu) and the *Guidelines for the Treatment of Marine Mammals in Field Research* published by the Society for Marine Mammalogy (www.marinemammalscience.org).

Chemical sedation was performed similar to previous studies [36], [48]–[51]. Sedation was achieved using an initial intramuscular injection of Telazol (tiletamine and zolazepam) at a dose of approximately 1.0 mg kg^-1^. Intravenous access for anesthetic administration and blood sampling was via the extradural vessel using an 18 G, 3.5-inch needle or catheter. Intravenous doses of ketamine (0.25 - 1 mg kg^-1^) and diazepam (1–5 mg) were administered as needed to maintain immobilization (all drugs from Fort Dodge Laboratories, Ft. Dodge IA). Sedated procedures were performed under light anesthesia (Plane 1) and animals were eupnic throughout. A summary of the chemical doses used is provided in Table 2.

**Table 2:** The anesthetic doses used during selected procedures.

Study Group	mass		induction dose				ketamine				diazepam			
	kg		mg		mg/kg		mg		mg/kg		mg		µg/kg	
**sedated weanling**	106	(22)	58.0	(11.0)	0.55	(0.10)	630	(73)	5.94	(0.69)	4.6	(3.9)	4.36	(3.72)
**sedated yearling**	111	(11)	64.3	(11.0)	0.58	(0.10)	594	(110)	5.35	(0.99)	12.0	(2.9)	10.81	(2.59)
**early weaning**	114	(17)	50.0	(0.0)	0.44	(0.00)	674	(194)	5.91	(1.70)	2.0	(2.3)	1.75	(1.99)
**late weaning**	94	(14)	45.0	(6.8)	0.48	(0.07)	372	(100)	3.96	(1.06)	0.3	(0.7)	0.32	(0.71)
**early lactation**	536	(33)	219.0	(22.5)	0.41	(0.04)	2200	(262)	4.10	(0.49)	42.5	(24.1)	7.93	(4.50)
**late lactation**	374	(50)	180.0	(12.6)	0.48	(0.03)	2286	(507)	6.11	(1.36)	14.3	(10.6)	3.82	(2.83)
**molting female**	311	(21)	168.3	(32.0)	0.54	(0.10)	1675	(301)	5.39	(0.97)	10.2	(7.1)	3.28	(2.28)
**Grand Mean**					0.496	(0.062)			5.25	(0.88)			4.61	(3.62)

The total drug doses are reported as the mean and (sd). The induction dose was equal parts tiletamine and zolazepam—values shown are for each. For each agent the total dose, in mg or µg, and mass-specific doses are reported. The induction dose was administered in a single intramuscular injection; ketamine and diazepam were administered intravenously periodically over 3–3.5 hours of sedation. Data of anesthetic doses for adult males are not reported.

Initial blood sampling occurred 10–15 min after Telazol administration (5–10 minutes before the onset of the EGP measurement). Following initial blood sample collection, the glucose tracer was administered and blood samples were collected periodically for 3 hours; these serial samples were used for the EGP measurement and assessment of hormone concentrations. Immediately upon returning to the lab, blood samples were centrifuged at 800 *g* and 4°C, the plasma or serum collected and was stored at -80°C until further analysis. The injection of glucose tracer was defined as time zero and subsequent sample times are reported relative to tracer injection.

### Field Sedated Group

Five weaned pups (4 female and 1 male) were measured early and late in their post-weaning fast in a paired sampling design. Adult females were measured early and late in the lactation period and after the completion of molting in a mixed sampling design. Five adult males were studied early and late in the breeding season and after molting in an unpaired (cross-sectional) sampling design. The duration of the blood sampling period was typically 180 minutes but limited to 150 minutes in adult males. Lactation and fasting durations were determined by marking and monitoring seals daily during the breeding season and throughout postweaning fasts. Molting seals were studied after the completion of molting, determined by pelage coloration.

### Handling Manipulated Group

Experimentally manipulated weanlings (2 female and 3 male) were studied at the Año Nuevo rookery in two states—chemically sedated and physically restrained. These weanlings had been weaned 3–4 weeks prior to measurements, determined by monitoring mother-pup pairs during the breeding season. To minimize diurnal variability these measurements were made midday, between 0900-1300. We varied the order of treatment procedures among animals, with three animals receiving sedation on the first day of handling and two sedated on the second day. The following morning weanlings underwent the second sampling procedure. The physical restraint measurement was conducted by placing the subject on a specially designed restraint board with nylon straps to minimize animal movement. Chemical sedation was performed as described above and weanlings were kept in custom-made aluminum transport cages between the two procedures.

Yearlings (4 female and 3 male) were captured from the same field location, placed in transport cages, and transported by truck to the animal holding facility at Sonoma State University, Rohnert Park, CA, for study. The cage dimensions were approximately 0.6×0.6×2.25 m, large enough for juvenile seals to move freely while minimizing their ability to turn around. Yearlings were captured after the completion of molting, in May–June, and had an estimated fasting duration of 3–4 weeks, similar to that of the weanlings. Yearlings were measured in two handling states—chemically sedated and unrestrained but confined within a transport cage. EGP was measured under chemical sedation on the same day as capture. At the end of the procedure an indwelling catheter (16 G×20 cm, MILA# 1610) was inserted into the extradural vessel and a 60″ extension tube filled with saline was attached to the catheter and sealed with a cap. The animal was allowed to recover from sedation overnight and the catheter was maintained patent by a periodic saline flush. The following morning we quietly performed a second measurement in the unrestrained yearling confined within the transport cage with minimal disturbance to the seal. Tracer injection and blood sampling were conducted as before but via the catheter and extension tube. The degree of alertness varied between individual study animals and over time during the measurement. One unrestrained EGP measurement was not made due to loss of catheter patency.

### Hormone Analyses

To assess the stress response to the different animal handling methods, cortisol and epinephrine concentrations were measured from blood samples drawn immediately prior to and approximately every 30 min during the EGP measurement. Epinephrine concentrations were only measured in the handling manipulated seals. Both hormones were measured using commercially available radioimmuno assay (RIA) kits (Siemens cortisol coat-a-count kit TKCO2; and Alpco epinephrine double-antibody kit 17-EPIHU-R50, Salem NH). The cortisol kit has previously been validated in this species [36], [44]. The epinephrine kit was validated for this study using serially diluted elephant seal plasma and significant parallelism with the standard curve was observed within the range of concentrations detected in this study. Average CV's for the cortisol and epinephrine assays were 2.9 and 3.1%, respectively. Several samples did not contain detectible levels of epinephrine. These non-detectible values were assigned the detection limit of the kit, 55 pM, for statistical analysis.

To assess the total hormone response during the measurement period, we calculated the area under the curve (AUC) over time by summing the areas under the hormone vs time polygons between sampling points and standardized for procedure duration by dividing by the total duration of the sampling period (e.g. 180 min).

### Plasma Glucose & EGP

**Figure 2:**
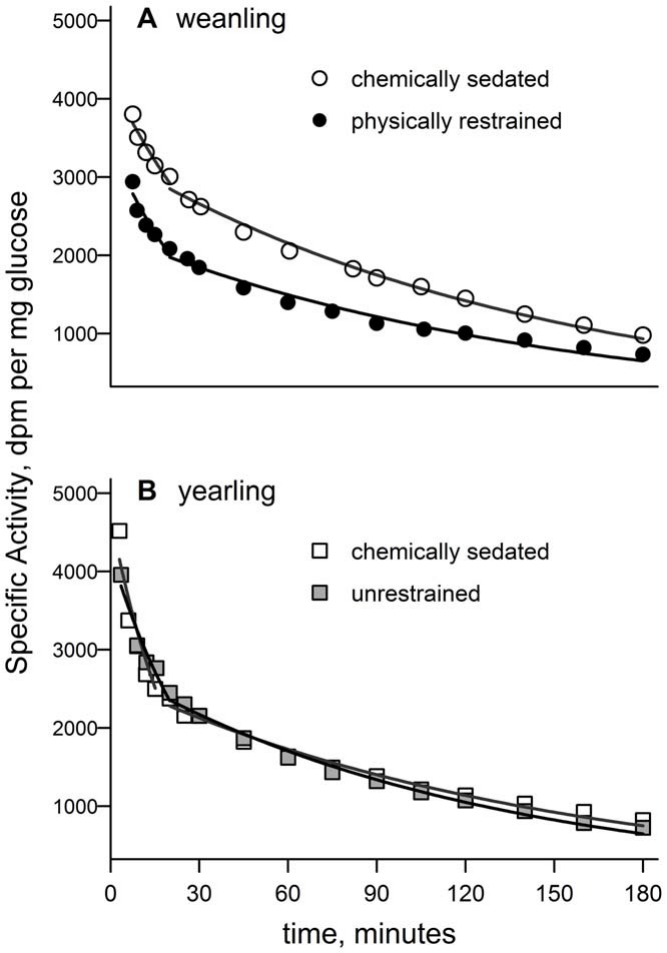
Example of [6-^3^H]glucose clearance curves used to calculate endogenous glucose production (EGP). Curves are shown for one weanling and one yearling with and without the use of anesthetic agents using the same tracer dose. The lower specific activity observed in the weanling under physical restraint compared to chemical sedation indicates increased dilution of the label from higher rates of EGP under physical restraint. Equivalent ^3^H doses were administered to each seal, 100 µCi. dpm—disintegrations per minute.

For all groups a bolus injection technique and non-compartmental model were used to measure the rate of tracer dilution [52]. A description of EGP measurement methods for field sedated animals may be found in Champagne et al [36], [53]. The rates of EGP for the field sedated animals have been reported previously: weaned pups [36], adult females [53], and adult males [54]. For the handling manipulated group, each seal was administered 100 µCi of [6-^3^H]glucose via the extradural vein. After injection, blood samples were serially drawn for 3 hrs. Typically 13 - 16 samples were taken over the measurement period, although performing these procedures without the use of immobilizing chemicals dictated some variation in the precise sampling intervals among study animals. The specific activity of [6-^3^H]glucose was determined as described in [36], [53]. Briefly, plasma samples from each sample time point were thawed and deproteinated using barium hydroxide and zinc sulfate (each 0.3 N, Sigma-Aldrich, St Louis, MO). Deproteinated plasma was then passed through ion exchange columns; the eluant was collected, dried, and reconstituted in water. The glucose concentration of each reconstituted sample was measured using a glucose analyzer (YSI 2300, Yellow Springs, Inc, Yellow Springs, OH). Reconstituted samples were then aliquoted in duplicate, scintillation cocktail was added and samples were counted on a liquid scintillation counter (Beckman LSC 6500; Fullerton, CA). [6-^3^H]glucose specific activity was calculated for each sample as the disintegrations per minute (dpm) per mole glucose. The rate of glucose production was measured by the dilution of isotopically labeled glucose by unlabeled glucose produced over time and was determined by dividing the dose injected by the area under the clearance curve
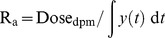
Where R_a_ is the rate of appearance of unlabeled glucose, Dose_dpm_ is the radioactivity of the injected tracer in disintegrations per minute (dpm), and y(*t*) is the exponential function describing the decay of the tracer specific activity with respect to time [52]. Two exponential functions were fit to the clearance curve by maximizing the r^2^ value for each function; curve-fitting and integration were performed using *Mathematica* (Wolfram Research, Champaign, IL). The typical inflection point occurred 20 minutes post glucose administration. Mean r^2^ values were 0.91 for the initial tracer dilution curve and 0.98 for the latter turnover curve (before and after the inflection point, respectively). Representative glucose dilution curves with and without the use of anesthetic agents are shown in Figure 2. The volume of the tracer administered to each study animal was determined by gravimetric calibration of the injection syringe. In this model of glucose kinetics, the rate of tracer dilution, R_a_, is equal to EGP and to the total uptake by all body tissues. Plasma glucose concentration was measured from blood samples drawn at the onset of the EGP measurement for all study animals and approximately every 30 minutes during the EGP measurement in the field manipulated study animals using a glucose analyzer (YSI 2300, Yellow Springs Instruments). In these study animals, glucose concentrations were averaged across the sampling period as an index of circulating glucose concentration during the procedure.

### Data Analysis

Paired t-tests were used to detect differences between groups of paired individuals. To test for significant differences among groups of unpaired individuals a linear mixed effects model with seal ID as a random effect was used, followed by post-hoc tests to compare between groups. In each instance we tested the full model, including interaction terms; when the interaction terms were not significant they were removed from the model. To investigate changes in hormone concentrations during the sampling period we performed repeated-measures analysis using a linear mixed model with sample time and study group as fixed effects and seal ID as a random effect; when differences among sample times were detected we tested for differences from initial concentration using LSD post-hoc tests. There was no apparent order effect of procedure day between weanlings physically restrained on day one versus day two so this factor was not included in analyses. Statistical tests were performed using R (version 2.11.1, R Development Core Team, www.R-project.org) and JMP ver 9 (SAS institute, Cary NC).

## Results

### Cortisol Response

**Figure 3:**
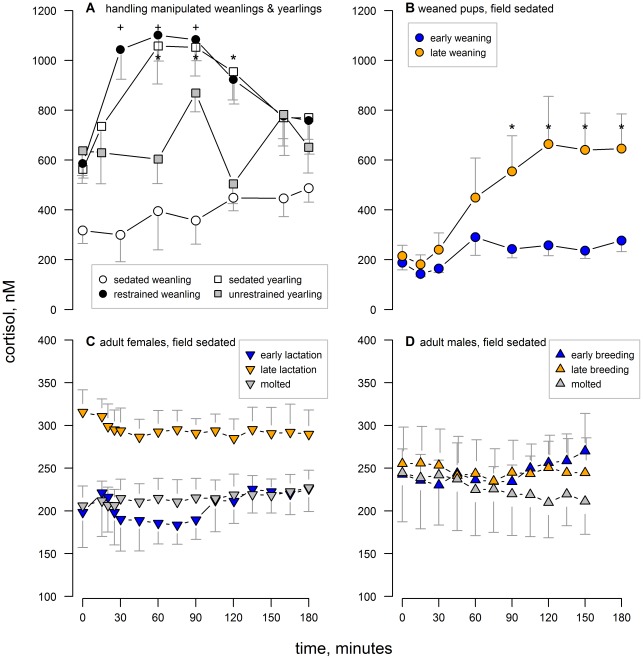
Cortisol concentrations during handling. The average cortisol concentration at each sample time within each study group; error bars represent standard errors. Note that the y-axis scales are different between the top and bottom graphs. RM ANOVA followed by pairwise post-hoc t-tests were used to test for significant differences from initial cortisol concentration. **A**) Handling manipulated group—physically restrained weanlings and chemically sedated yearlings showed increased cortisol levels during sampling. “+” and “*” indicate significant differences from initial (time = 0) cortisol value for physically restrained weanlings and chemically sedated yearlings, respectively (pairwise post-hoc t-test, p<0.05). **B**) Weaned pups early and late in post-weaning fast—late in the post-weaning fast, pups showed increased cortisol concentrations after 100 minutes of chemical sedation. “*” indicates significant difference from initial cortisol value (pairwise post-hoc t-test, p<0.05). **C**) Adult females early and late in lactation and after molting and **D**) adult males early and late in the breeding season and after molting. There was no significant difference in cortisol concentration with sample time among the adult samples.

The average cortisol concentrations at each sampling point and each study group are shown in Figure 3. Only a few treatment groups showed changes in cortisol concentration with sampling time. Within the chemically sedated weanlings there was no significant change in cortisol concentration with sample time (F_6, 20.7_ = 0.9, p = 0.53) but physically restrained weanlings had elevated cortisol concentration during much of the measurement period (F_6, 23_ = 5.2, p = 0.002; Figure 3A). There was no significant change in cortisol concentration with sample time in unrestrained yearlings (F_6, 23.0_ = 1.4, p = 0.26) whereas it was elevated under chemical sedation (F_6, 27.6_ = 3.5, p = 0.01; Figure 3A). Among weaned pups sedated in the field, cortisol concentration did not significantly change with sample time early in the post-weaning fast (F_7, 28.0_ = 2.1, p = 0.07) whereas there was a significant change late in the fast (F_7, 28.0_ = 4.36, p = 0.002; Figure 3B). Among adult females, cortisol concentration varied by study group (F_2, 29.5_ = 227.7, p≤0.001) but there was no effect of sample time on cortisol concentration (F_14, 213_ = 1.0, p = 0.51; Figure 3C). Within adult males there was no difference in cortisol concentration among study groups (F_2, 12_ = 0.1, p = 0.9) nor did cortisol vary with sample time (F_10, 120_ = 0.4, p = 0.95; Figure 3D).

**Figure 4:**
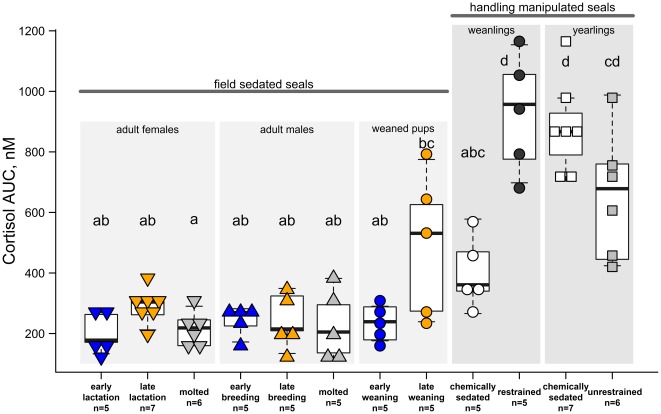
Cortisol AUC value for each study group. The total cortisol present during the sampling period (cortisol AUC) for each study group; symbol and color coding matches that of Figure 3. Groups without overlapping letters were significantly different (p<0.05). See text and Table 1 for additional descriptions of study groups. Central horizontal lines indicate median of each group; whiskers extend to data points within 1.5 times the interquartile range from each box.

Cortisol AUC values varied among study groups (F_11, 46.5_ = 25.2, p≤0.001; Figure 4). The least cortisol response to handling was observed in animals chemically sedated in the field while the greatest was in physically restrained weanlings and yearlings that were transported to the lab for study. Physical restraint increased cortisol levels in weanlings; both initial and AUC values were greater under physical restraint than chemical immobilization (paired t = 3.0 4.8; p = 0.04, 0.008, respectively). In yearlings, cortisol levels were not different between the sedated and unrestrained states for either initial or AUC values (paired t-tests, p>0.1).

### Epinephrine Response

**Figure 5:**
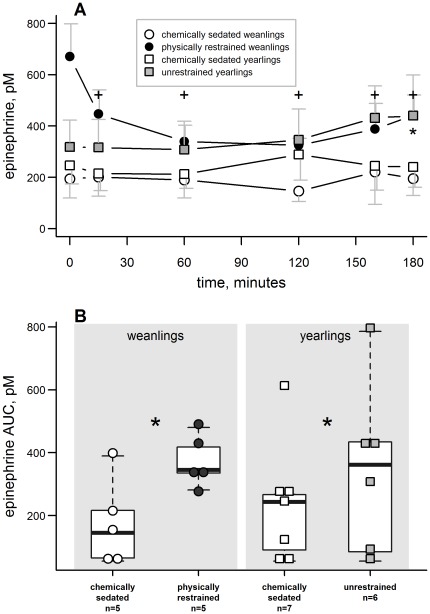
Epinephrine concentration in handling manipulated seals. **A**) Epinephrine concentrations were generally stable during procedures except in physically restrained weanlings. These restrained seals had elevated epinephrine concentrations at the beginning of the procedures; “**+**” and “*” indicate significant difference from initial epinephrine concentration in physically restrained weanlings and unrestrained yearlings, respectively (pairwise post-hoc t-test, p<0.05). Error bars are standard errors. **B**) The lowest epinephrine AUC values occurred while study animals were chemically sedated in both weanlings (paired t = 2.9, p = 0.045) and yearlings (paired t = 2.9, p = 0.03). Central horizontal lines indicate the median of each group; whiskers extend to data points within 1.5 times the interquartile range from each box.

Epinephrine was only measured in the handling manipulated group. The average epinephrine concentrations during these procedures are shown in Figure 5A. Epinephrine concentration varied with sample time (F_5, 88.3_ = 2.4, p = 0.04). There was no effect of study group (p = 0.17) but the group-by-sample time interaction was significant (F_15, 88.3_ = 2.0, p = 0.02). There was no change in epinephrine concentration with sample time in sedated weanlings (LSD post-hoc tests, p>0.05) but sample time had a significant influence on epinephrine concentration in physically restrained weanlings. Within this group the initial epinephrine concentration was different than that from any other sample time (LSD post-hoc tests, p<0.05). In contrast, there was no significant difference in epinephrine concentration with sample time in sedated yearlings but there was in the unrestrained group, though only the 180 min sample was different from time zero (LSD post-hoc test, p<0.05).

Epinephrine AUC values varied among study groups (F_3, 12_ = 5.8, p = 0.01; Figure 5B). Physical restraint resulted in higher initial epinephrine and AUC values in weanlings (paired t = 3.6, 2.9, p = 0.02, 0.045, respectively) and unrestrained yearlings had higher epinephrine AUC values than during chemical sedation (paired t = 2.9, p = 0.03; Figure 5B) but the yearlings' initial epinephrine concentrations were not different between the sedated and unrestrained states (paired t-test, p>0.6).

### Glucose Metabolism During Handling

**Figure 6:**
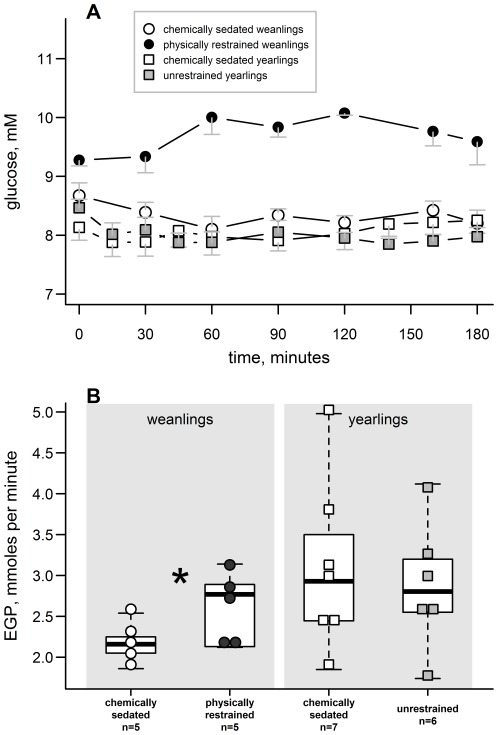
Glucose response in handling manipulated seals. **A**) The average glucose levels in physically restrained weanlings during the EGP measurement were significantly higher than the other groups (F_3, 11_ = 15.5, p<0.001). Error bars represent standard errors. **B**) Physical restraint significantly increased EGP (*) in weanlings (paired t = 3.1, p = 0.04) but there was no difference in EGP between chemically sedated and unrestrained yearlings (paired t-test, p>0.05). Central horizontal lines indicate the median of each group; whiskers extend to data points within 1.5 times the interquartile range from each box.

Plasma glucose concentrations were measured from samples taken periodically during the EGP measurement in handling manipulated animals (Figure 6A). Both the initial plasma glucose concentrations and average levels during the EGP procedure varied by study group (initial concentration: F_3, 11.9_ = 6.9, p = 0.006; average levels: F_3, 11_ = 15.5, p≤0.001). Glucose concentration was higher during physical restraint than during chemical sedation, both the initial concentrations and average levels throughout the sampling period (paired t = 4.7, 8.2; p = 0.018, 0.004, respectively). There was, however, no difference in plasma glucose level between chemically sedated and unrestrained yearlings (paired t-test, p>0.6).

EGP was 20% higher in weanlings under physical restraint compared with chemical immobilization (paired t = 3.1, p = 0.04, Figure 6B). There was no difference in EGP between sedated and unrestrained yearlings (paired t-test, p>0.2). Additionally, there was no difference between yearlings and weanlings of any group (F_3, 12_ = 2.2, p = 0.14; individual variation accounted for 74% of the variability in EGP). The rates of EGP for field-sedated animals have been reported elsewhere [36], [53], [54].

**Figure 7:**
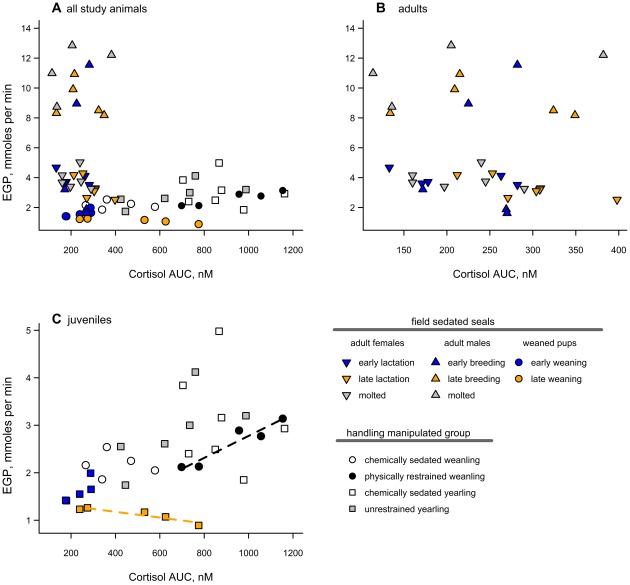
Relationship between EGP and cortisol levels. EGP did not vary with cortisol release among all study groups (F_1, 14.7_ = 1.0, p = 0.33). Note that the axes are not consistent between graphs, **A**) all study animals shown; **B**) adults, and **C**) juveniles, because the handling manipulated seals had a much larger range in cortisol AUC values than did field sedated seals. Dashed lines in **C**) indicate significant trends within the physically restrained and late weaning groups alone.

There was no relationship between EGP and cortisol AUC when accounting for body mass and study group (F_1, 14.7_ = 1.0, p = 0.33; Figure 7). Within physically restrained weanlings alone, however, there was a trend toward increased EGP with cortisol AUC values—in a multiple regression analysis of EGP by cortisol AUC and mass (full model: F = 38.5, p = 0.025; effect test for cortisol AUC: F = 71.3, p = 0.014; Figure 7C). Similarly, there was no correlation between EGP and epinephrine AUC values in a mixed-model analysis of EGP with study group, mass, and epinephrine AUC as predictors and seal as a random effect (F_1,16.5_ = 0.03, p = 0.8).

## Discussion

The method of handling had a significant influence on cortisol release and metabolism in northern elephant seals. Extended sedation is necessary to conduct metabolic measurements such as the EGP measurements described here, as well as other measurements including glucose tolerance tests [40], [55] and measures of lipolysis [38]. This study did not detect a cortisol response during extended sedation in adult northern elephant seals. Physical restraint caused increases in circulating cortisol, epinephrine, and glucose concentrations as well as increased EGP in weanlings. Transport appeared to sensitize seals to further manipulation as chemically sedated yearlings displayed a significant cortisol response, similar to that of physically restrained weanlings. The cortisol response in yearlings, however, was not associated with increased plasma glucose concentration or increased rates of EGP. These findings are similar to reports in other species, where sedation reduced or ameliorated the stress impacts of handling [56]–[59].

### Hormonal Response to Handling

Sustained physical restraint led to a marked stress response. Cortisol concentration increased from an initial level of 586 nM to over 1000 nM during physical restraint. During chemical sedation cortisol concentration remained steady throughout procedures in nearly all study groups. In order to study unrestrained seals, yearlings were first captured and transported to the lab before any measurements were made. These yearlings displayed cortisol and epinephrine responses similar to that of physically restrained weanlings. This contrasted with the response of animals sedated in the field. Regrettably, we did not sample yearlings in the field and there are no published data on cortisol concentration in molting northern elephant seal yearlings. However, Kelso [60] conducted a study of 40 yearlings over two years (in 2008 & 2009) during their annual fall haul-out and reported cortisol concentrations of 223 (s.d. 26) and 260 (s.d. 29) nM at the beginning and end of fasting, respectively. These values were significantly lower than cortisol concentrations of yearlings measured in this study (F_3, 53_ = 17.0, p≤0.001) which had similar fasting durations but were sampled in different seasons. The lower cortisol levels reported in yearlings measured in the field suggest that cortisol concentrations increased during transport and were elevated by the time we collected an initial blood sample. Cortisol concentrations increased further during chemical sedation in yearlings. The similarity in cortisol responses between physically restrained weanlings and chemically sedated yearlings suggests an acute response due to capture and transport. By the following morning, cortisol concentrations in yearlings held at the lab returned to their earlier levels but these were higher than reported values measured from animals sedated in the field. These patterns suggest that transport may be inherently stressful [61], despite the apparent tolerance of northern elephant seals to this type of handling and transport.

The timing of the cortisol release was similar in physically restrained weanlings and sedated yearlings (Figure 3A). Peak cortisol levels occurred at 30–60 min and declined after 90 min in both groups. These findings are similar to those of Engelhard and co-authors [57] who reported increased cortisol levels in southern elephant seal pups, *M. leonina*, during 45 min of physical restraint. Both the absolute cortisol concentration and the timing of the response to physical restraint were similar between the two studies. Investigations in grey seals, *Halichoerus grypus*, also detected increased cortisol levels with handling and restraint, in weaned pups [62] and adult males [63]. Grey seal pups had increased cortisol within ten minutes of initial handling whereas, in adult males, cortisol levels began to plateau after 30 minutes of continued restraint. In the present study, the sampling period was prolonged and we identified a peak and subsequent decrease in cortisol level while seals were still under physical restraint. The magnitude of the cortisol response was much greater in northern elephant seals—peak levels were over 1000 nM in physically restrained elephant seals, compared with ∼100 nM in grey seal weanlings and less than ∼480 nM in adult grey seals [62], [63].

Adult elephant seals displayed remarkably stable cortisol levels during 2.5–3 hrs of chemical sedation (Figure 3C and D). If the initial handling or anesthetic induction caused a substantial cortisol release, we would expect to find declining cortisol concentrations during the subsequent three hours of sampling under sedation. The stable cortisol concentrations observed suggest that there was not a cortisol release in response to typical sedation procedures and cortisol concentrations measured under these conditions are near baseline levels. Cortisol concentrations closely match those reported for southern elephant seals sedated using similar methods during lactation [57]. Engelhard et al did, however, detect a small but statistically significant increase in cortisol concentration ∼23 minutes after induction. While not statistically significant, data from early lactation and molted females in this study do show a similar trend (see Figure 3C). However, when sampling for 180 min vs 45 min in Engelhard et al, the parabolic trend in the data during the first 30 minutes appears even less substantial. There is sizeable evidence that the stress response is suppressed during lactation in several species [64]–[66]. Engelhard and co-authors therefore cautioned that the mild cortisol response observed during lactation in southern elephant seals may be a blunted indicator of the true level of stress [57]. This study included non-lactating, recently molted females and found no difference in the cortisol response between lactating and molted northern elephant seals. Rather, cortisol concentration increased during lactation (Figure 3C). In a similarly designed study in rhesus macaques, *Macaca mullata*, Maestripieri et al [67] found no difference in the cortisol response between lactating and non-lactating females; furthermore, they questioned the ubiquity of the suppression of stress response during lactation. One reason postulated for the suppression of a stress response during lactation is the importance of maintaining energy expenditure toward milk production vice other expenditures [66]. The energetic and nutrient constraints of extended fasting during elephant seal haul-outs, especially that of limited protein degradation, may favor a suppressed stress response in this species; although molting is substantially less energetically expensive than lactation [68]. Elephant seals are recognized for their tolerance to mild stressors and to human disturbance [69], [70]. The energy and water flux constraints on fasting elephant seals may contribute to their nominal response to mild perturbation.

As might be expected, epinephrine concentrations were higher in physically restrained than chemically sedated seals, indicating an acute stress response from restraint. Physically restrained weanlings exhibited an epinephrine release with the onset of restraint as initial concentrations were high and subsequently decreased in the first hour of handling. Unrestrained yearlings only showed elevated epinephrine concentrations at the very end of the procedure. Transient disturbances during sampling probably caused elevated epinephrine values toward the end of the procedure when seals were aware of the researcher's presence and their tolerance to disturbance may have decreased with time. The higher epinephrine AUC values also suggest that these unrestrained yearlings exhibited a greater catecholamine release than sedated seals.

### Handling Effects on Glucose Metabolism

Circulating plasma glucose concentration was higher in physically restrained weanlings than in other study groups but only by ∼7% (Figure 6A). This elevation was in addition to baseline glucose levels that are naturally higher than typically observed in mammals [71], [72]. A similar study did not detect differences in circulating glucose concentrations among suckling pups exposed to various handling intensities—pups handled from one to five times during a 21 day sucking period [57].

Increased plasma glucose levels were concomitant with increased EGP in weanlings (Figure 6). Among yearlings, there was a high degree of individual variability in the rates of EGP, with no significant difference between sedated and unrestrained states (Figure 6B). Unlike physically restrained weanlings, the increased cortisol levels observed during chemical immobilization in yearlings were not associated with higher circulating plasma glucose concentrations or rates of EGP. The rates of EGP reported here are similar to those previously reported in this species [36] as well as harbor, *Phoca vitulina* [73] and grey seals [39]. Typically EGP increases with exercise [74], [75] and physically restrained seals were often agitated and struggled periodically during measurements. While this was not a steady exercising regime, the increased activity under physical restraint was probably responsible for much of the increase in EGP.

### Psychoactive Chemicals and their Effect on Carbohydrate Metabolism

We measured EGP in young elephant seals with and without the use of a frequently-used combination of chemical agents—dissociative anesthetics (phencyclidines tiletamine and ketamine) and benzodiazepine sedatives (zolazepam and diazepam). The use of psychoactive chemicals certainly has the potential to disturb glucose regulation by the CNS and there is evidence for CNS regulation of fuel balance and glucose homeostasis [76]–[78]. Each chemical's target receptor and specificity will influence its effect, if any, on the regulation of fuel use. Ketamine, for example, has been shown to increase oxygen uptake of the CNS [79] potentially increasing glucose use by the brain. We detected lower rates of EGP in chemically sedated weanlings, compared to those being physically restrained. This difference was probably due to greater physical exertion in the restrained subjects. We found no detectable difference in rates of EGP between sedated and unrestrained yearlings, suggesting that this combination of chemical agents does not have a substantial effect on whole-animal carbohydrate metabolism in this species.

Previous studies on the effects of ketamine on carbohydrate metabolism have been equivocal and show species-specific variation. Within primates, Castro et al. [80] found no effect of ketamine on plasma glucose concentration as well as several hormones, including insulin, in long-tailed macaques, *Macaca fascicularis*. Similarly, Kemnitz and Kraemer [81] found no effect of extended duration (2 hours) ketamine sedation on fasting glucose or insulin and there was no effect on the physiological responses to hyper or hypo-glycemia in rhesus monkeys, *M. mulatta*. Alternatively, Lehmann, et al. [82] found that the administration of ketamine influenced glucose metabolism by lowering plasma glucose, inhibiting insulin secretion, and increasing glucagon and cortisol levels in baboons, *Papio hamadryas*. In rats the combination of ketamine and xylazine increased blood glucose levels while fasting and profoundly so in fed rats [83]; there was, however, no effect of ketamine alone. The ketamine dose performed by Saha and co-authors was 50 mg ketamine (kg · hr)^-1^. This dose far exceeds that commonly administered to elephant seals; the average dose in a typical study of weaned elephant seal pups, for example, is 2 mg ketamine (kg · hr)^-1^, see Table 2. The initial induction using Telazol in this study allows for decreased ketamine use; nonetheless, the ketamine dose differential remains striking and their findings may not be applicable to the chemical administration protocol in the present study or other similar field-use protocols.

There are several studies on the effect of ketamine on hormone concentration, e.g. [84], [85]. N-methyl-D-aspartate (NMDA) receptors are found within the anterior pituitary and are involved in the regulation of hormone release from the hypothalamic-pituitary-adrenal (HPA) axis [86]–[88] and it appears that the NMDA receptor antagonist ketamine does impact HPA axis function at some level. Data are conflicting, however, regarding ketamine's effect on circulating glucocorticoid concentration. Studies of low-dose ketamine infusion (0.3–0.5 mg kg^-1^) found that cortisol levels significantly increased [85], [89] but results may not apply to anesthetic doses of ketamine. In fact, the study of Saha et al [90] discussed above found decreases in both adrenocorticotropic hormone and corticosterone in rats under higher doses of ketamine in combination with xylazine. To distinguish between the effects of anesthetic agents and effects of capture and handling, measurements on captive pinnipeds habituated to human contact are needed. However, the combination of phencyclidines and benzodiazepines used in this study does not appear to influence cortisol concentration in elephant seals chemically sedated in the field.

### Glucose Production Was Not Correlated with Physiological Cortisol Release in Elephant Seals

Among all 12 study groups, there was no relationship between EGP and cortisol levels (Figure 7A). Although cortisol is known to stimulate glucose production in mammals [34], [91], this lack of correlation in elephant seals is not necessarily surprising. Previous studies in northern elephant seals have failed to detect relationships among regulatory hormones and measures of circulating metabolites and fuel use [38], [40], [53]. Among adults, cortisol variability was small compared with the variation in EGP. Within the physically restrained weanlings alone, however, EGP was positively correlated with cortisol AUC value (Figure 7C). This suggests that large releases of cortisol in response to a strong stressor like physical restraint may have gluconeogenic effects in fasting elephant seals. To determine the direct influence of cortisol (as well as insulin and glucagon) on EGP in seals, glucose-clamped hormone challenge experiments will need to be performed in combination with measurements of metabolic flux. Nevertheless, the current data suggest that normal variability in cortisol concentration during chemical sedation does not have a significant influence on EGP measurements in elephant seals. Furthermore, the lack of difference in rates of EGP between yearlings with and without the use of anesthetic chemicals suggests that the standard chemical sedation techniques used do not have a significant effect on carbohydrate metabolism in this species.

### Conclusion

Capture or physical restraint resulted in a marked stress response whereas chemical sedation mitigated an increase in hormone stress markers during handling in elephant seals. When potential impacts of capture stress were evaluated by measuring one metric of whole-animal metabolism, EGP, we found that physical restraint increased EGP. Chemical sedation, however, did not significantly influence the rate of EGP. This study demonstrated that some species, such as northern elephant seals, can be sedated for prolonged periods for metabolic studies without significant stress responses or alterations in glucose metabolism. Manipulation, including physical restraint and transport, induced increases in stress hormone levels; these increased levels, however, did not necessarily result in increased rates of EGP. Our findings suggest that for measurements that may be influenced by stress responses, even in species apparently tolerant to disturbance such as northern elephant seals, it is preferable to conduct measurements in the field under chemical sedation, rather than by physical restraint or animal capture followed by transport to a laboratory for investigation.
